# “It's Like a Kick in the Teeth”: The Emergence of Novel Predictors of Burnout in Frontline Workers During Covid-19

**DOI:** 10.3389/fpsyg.2021.645504

**Published:** 2021-05-25

**Authors:** Rachel C. Sumner, Elaine L. Kinsella

**Affiliations:** ^1^Health, Environmental Responsibility & Action (HERA) Lab, School of Natural & Social Sciences, University of Gloucestershire, Cheltenham, United Kingdom; ^2^Research on Influence, Social Networks, & Ethics (RISE) Lab, Department of Psychology, Centre for Social Issues Research, Health Research Institute, University of Limerick, Limerick, Ireland

**Keywords:** burnout, collective action, Covid-19, CV19 Heroes, frontline workers, heroes, occupational stress, social solidarity

## Abstract

The context of Covid-19 has offered an unusual cultural landscape for examining how workers view their own position relative to others, and how individuals respond to prolonged exposure to workplace stress across different sectors and cultures. Through our recent work tracking the well-being of frontline workers in the UK and Ireland (the CV19 Heroes project), we have uncovered additional psychological factors that have not been accounted for in previous models of occupational stress or burnout. In recent months, frontline workers have worked to protect the community from the threat of SARS-CoV-2 and, simultaneously, have evaluated their perceptions of collective efforts of others as either congruent or incongruent with collective goals (e.g., lowered mortality and morbidity): we call this novel aspect *solidarity appraisal*. These frontline workers have been hailed as heroes, which we argue has led to the creation of an implicit psychological contract (*the hero contract*) between frontline workers and the public. Here, the heroes are willing to “go above and beyond” for the greater good, with the expectation that we (the public) do our part by adhering to public health guidelines. Where frontline workers perceive incongruence between the words and actions of others in working toward collective goals this drives negative affect and subsequent burnout. In this perspective article, we evaluate the cultural context of the pandemic in the UK and Ireland and suggest important socio-cultural factors that contribute to perceptions of solidarity, and how this may relate to burnout and worker welfare during and beyond the pandemic context.

## Introduction

Occupational stress has been long known to impact both physical and mental health in a variety of ways (Taris, 2016). From increased likelihood of cardiovascular disease (Kivimäki and Kawachi, 2015), metabolic syndrome (Chandola et al., 2006), and cardiovascular mortality (Kivimäki et al., 2002) to associations with chronic pain (Herr et al., 2015), there has been a great deal of work carried out to understand the specific correlates of stressful work on physical health and functioning. For mental health outcomes, certain concepts of mental ill-health exist solely because of the strains of work such as burnout (Maslach et al., 2001) and its associated impacts on traumatic stress (Galek et al., 2011), depression (Schonfeld and Bianchi, 2016), and anxiety (Koutsimani et al., 2019).

Working on the frontline during Covid-19 has necessitated great self-sacrifice on the part of all of those in these roles during the shifting social contexts of the pandemic since it began. From the earlier days of little testing and little protection, and during panic buying, to times of reports of non-compliance by the public and by notable figures (in the UK and Ireland, but also elsewhere in the world), through to displays of defiance and protest associated with pandemic denial or public health measure resistance, the workers have continued. Not surprisingly, much research has been done to understand the impacts of working on the frontline. Work carried out thus far mostly focuses on frontline healthcare workers, where severe mental health implications of this work have been described in the form of post-traumatic stress disorder, anxiety, depression, and somatic symptoms (e.g., Carmassi et al., 2020; Giorgi et al., 2020; Preti et al., 2020), but increased burnout and decreased resilience and well-being have also been described in work incorporating broad profiles of frontline workers (Sumner and Kinsella, 2021). Data from the latter project, known as the CV19 Heroes project—the CV19 Heroes Project^1^ which a key aspect was to understand burnout in the context of pandemic stress and occupational demand—has led to new theoretical developments presented here.

## Burnout in Context

Burnout has been well-studied over the years, most notably by Christina Maslach, whose authoritative work has been at the centre of developments within this field. Burnout is defined as being a psychological syndrome that is characterised by a progression of occupational stress, and is manifested in cynicism, exhaustion, and feelings of inadequacy or inefficacy (Maslach, 1993; Maslach et al., 2001). Conditions for burnout are said to be associated with a mismatch between the individual and their working environment, in any one of six domains: *workload, control, reward, community, fairness*, and *values* (Maslach and Leiter, 2008). While the concept of the social environment (or *community*) contributing to burnout has been well-studied, it focuses solely on social interactions and interplay *within* the organisational setting (Maslach and Leiter, 2008). Wider social and cultural influences on burnout have not yet been considered in any depth.

There are many occupations that are associated with higher instances of burnout amongst their workers (Leiter and Schaufeli, 1996; Taris et al., 2005). Many of these lines of work (frequently in helping roles) are reliant on engagement with the public such as healthcare workers, veterinarians, civil defence and emergency services (Felton, 1998; Ben-Zur and Michael, 2007; Platt et al., 2012). The stressful impacts of these types of work are cited as being related to an externally-situated control, such as with colleagues, customers/clients, or line managers (Taylor and Cooper, 1989; Glass and McKnight, 1996). Elements of the work within those professions require interdependence of action and, as a result, the control over outcomes is shared^2^. Within the Covid-19 pandemic, interdependence is critical. Frontline workers are reliant on the public to adhere to public health guidance to prevent the spread of the virus, thereby reducing their potential workload, or otherwise reducing the distressing conditions of their work. Importantly, in this context, we have seen evidence for the wider socio-cultural environment playing a key part in workers' feelings of reward from their efforts. We suspect that these broader influences on burnout play out in a range of professions. Veterinary practitioners, for example, may experience burnout due to client non-compliance resulting in animal suffering or need for prolonged or more aggressive treatment (e.g., Ballantyne and Buller, 2015; Moses et al., 2018). While the cognitive appraisal of a stressor and one's own resources to cope with it has been long-studied (e.g., Folkman, 1984; Folkman and Lazarus, 1984), the extent that other people in the broader social context (external to one's organisational setting) are appraised as working toward related goals when success is contingent on this has not been previously considered. We call this *solidarity appraisal* and believe it to be an important contributor to burnout in frontline workers and other demanding professions that require interdependence of action.

Recently, we explored the experiences of frontline workers in the UK and Ireland during the pandemic (Kinsella et al., In Press) using interviews from 38 frontline workers, with a balance across the UK and Ireland, and from different frontline sectors (i.e., health and social care, community supply chains, and civil defence). Our work uncovered aspects of perceived public action that many frontliners found deeply upsetting and difficult, which have theoretical implications for the psychology of burnout. Firstly, the perceptions of non-compliance with public health regulations. Participants noted their feelings of inadequacy and frustration at the behaviour of others (including known and unknown members of the public, and government officials), in reference to their perceived non-compliance with public health regulations, and minimisation or otherwise denial of the existence or severity of the pandemic. One participant explained:

Thinking about other people, it's like a kick in the teeth if you're a healthcare worker. We're doing all these efforts. Some of my colleagues didn't see their kids for 2, 3, 4, 5 weeks. One of the girls was working and her kid is on immuno-suppressants, so her little boy had to live with the grandparents for 6 weeks. She didn't see her kid and then come back out and see [them] going on the beach and demonstrating with no mask, it is like a kick in the teeth. It's infuriating for us.

A key factor of interest here is the extent that the worker appraises others in their own cultural context to engage in the required collective and co-operative action. We live in a world where we can witness with our own eyes but can also witness with the eyes of the population's citizens via news and social media. Within the pandemic, the world's attention has focused on the minutiae of the crisis as it has unfolded. Arguably, socio-cultural influences on burnout have become more important within the context of a 24 hour news cycle, and where accessibility to information and online interaction through social media is constant. Stories of tragedy, misfortune, or mishap have been a key focus of news media, and various cultural narratives have pervaded, including social rhetoric around “Covidiots” (Romain, 2020; Reicher and Drury, 2021). Much Western news and social media has often focused on images and stories of non-compliance, panic buying, conspiracy rhetoric, and legislative hypocrisy, which have been shared across the globe (e.g., Arafat et al., 2020; Islam et al., 2020; Lunn et al., 2020; Mevorach et al., 2021). Narratives surrounding sensationalised non-compliance in the public have wound up in unhelpful discourses of morality (Prosser et al., 2020), some of which have been instigated from central government in many countries (e.g., Forester and McKibbon, 2020; Liao et al., 2020; Zahariadis et al., 2020; Reicher and Drury, 2021). As a result, the frontline workers whose sacrifices have increased are witnessing a lack of solidarity that has been vastly magnified and prioritised over coverage of dutiful compliance or positive public health messages (Basch et al., 2020). This media coverage has also been shown to drive public behaviour during the pandemic (Gozzi et al., 2020), actually encouraging or, at the least, legitimising rule breaking, making the overall social contextual situation all the worse for frontline workers. Solidarity is, perhaps, particularly valuable in the frontline worker setting because it carries with it implicit connotations of respect, empathy, social justice, and reciprocity (Molm et al., 2007; Molm, 2010; Stavrova and Schlösser, 2015). One participant stated:

I think if people have the opportunity to go and, you know, see how, you know, walk a mile in someone else's shoes, you might realise just how difficult it is.

When viewed through this lens, it becomes clear the role that legislative leadership and the public have in influencing perceptions of solidarity and minimising the potential damage of the pandemic by adhering to public health guidance (van Bavel et al., 2020).

Our work also uncovered a second aspect of perceived public action that many frontliners found deeply upsetting and difficult: a perceived discrepancy between public words and public deeds. The rhetoric of frontline keyworkers being called heroes, particularly during the first surge where there were weekly “clap for heroes” evenings from the public was sharply at odds with perceptions of behaviour contravening public health guidance. The incongruence between words and deeds also has been apparent outside of the pandemic context. For instance, during the Olympic Games of 2012, the UK National Health Service (NHS) featured as a point of pride and celebration in the opening ceremonies, yet, at the same time, was controversially reorganised and staff wages were frozen (Burki, 2018)—a clear discrepancy between public/political rhetoric and action, which may have negatively impacted workers' well-being.

During the pandemic, perceived incongruence between words and actions may have been further amplified using the powerful cultural label, *hero*. Frontline workers have worked in extremely challenging environments for extended periods of time during the pandemic to help, protect, and save others. They have quite rightly been hailed as heroes due to their extraordinary displays of bravery, sacrifice, and compassion for others. However, there has been much critique levelled at this rhetoric for diminishing the sacrifices made by those on the frontline (with the thesis that as they are heroes, sacrifices are the expectation), as well as setting those labelled as hero up with unrealistic expectations of enduring resilience and strength (Hsin and Macer, 2004; Cox, 2020; Stokes-Parish et al., 2020). Recent work critiquing the use of the hero label for healthcare workers in the pandemic has also highlighted the need for reciprocity between frontline workers and the general public (Cox, 2020; Kinsella and Sumner, 2021). One participant emphasised this point:

But people wouldn't [follow public health guidelines] and then the next minute they're saying “you're heroes” and it's like “No, you [are] being stupid and creating work for us and putting us at risk.”

Frontline workers have not only had to deal with the challenge of trying to fulfil their role responsibilities contingent on the engagement of the public (interdependence of effort in attaining goals), but also to live up to these heightened expectations and stereotypes of being a hero (e.g., brave, strong, unwavering: Kinsella et al., 2015b). While there has been no research as yet examining hero labelling and burnout, the potential stress of unrealistic expectations from this label taps into the concepts of *workload* (by the public demands of the hero role exceeding their human capabilities), and *reward* (by reducing intrinsic reward and achievement as they may reasonably fail). This latter aspect is perhaps the most painful for frontliners as it may be inherently unavoidable—for healthcare workers they have faced the death of many patients as a result of Covid-19, and for supermarket workers they have struggled to keep produce available during high demand (Kinsella and Sumner, 2021).

**Figure 1:**
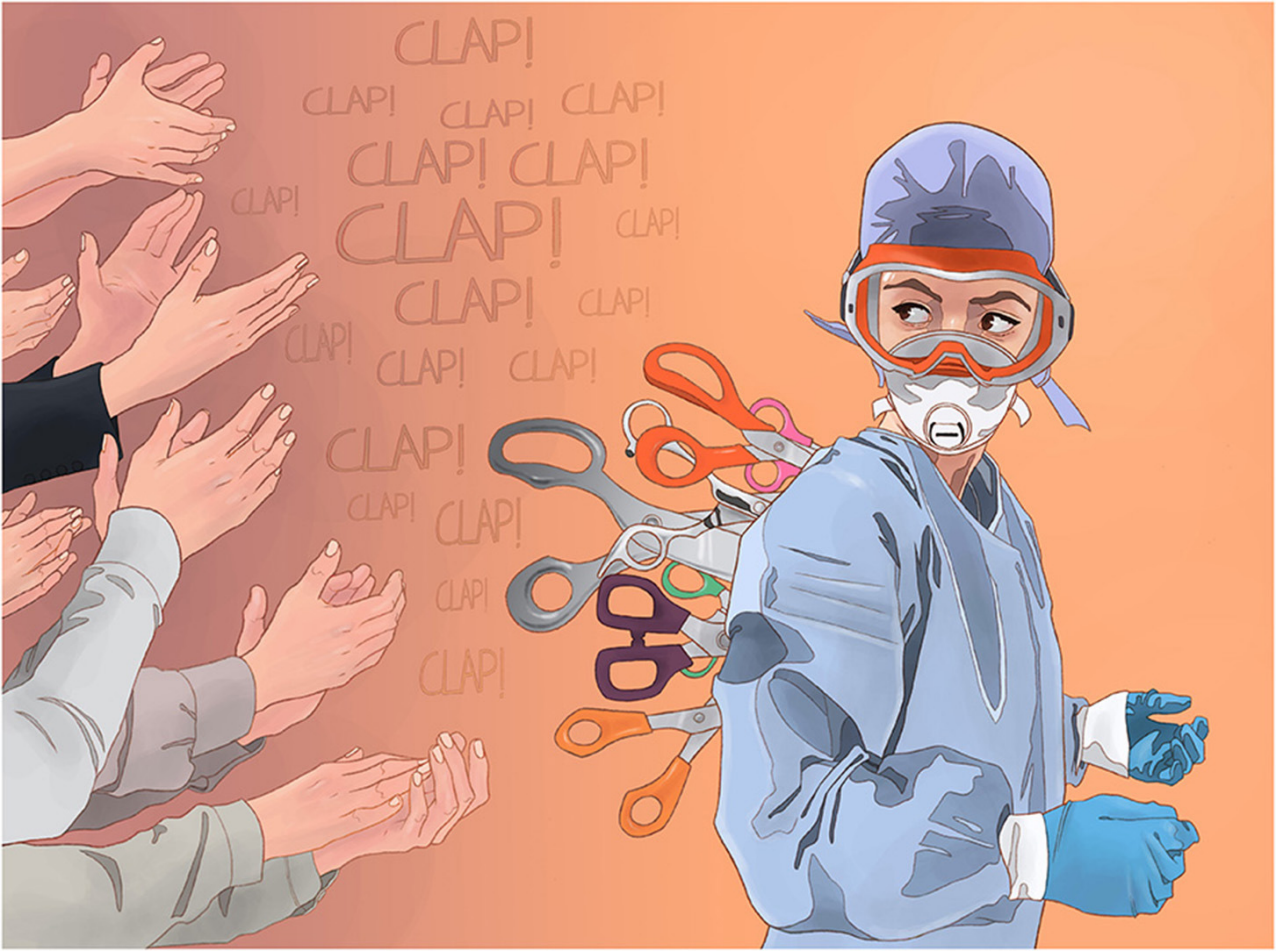
Luis Quiles' work “Expendable Workers” (ca. April 2020) depicting the contradiction in words and deeds perceived by frontline workers in Covid-19. Reproduced by kind permission of the artist.

A perceived discrepancy between words (hero label) and deeds (non-compliance, conspiracy) could be likened to the breaking of a psychological contract (Rousseau, 1989; Rousseau and Parks, 1993) (or *hero contract*)—where frontline workers were willing to go “above and beyond” for the greater good of society with an implicit agreement that others would adhere to public health advice. Breaches of psychological contract in the work context can lead to a variety of negative outcomes, including burnout (Jones and Griep, 2018). The hero contract is, therefore, a promise of conduct on the part of the person providing it—to behave in a way that supports the attribution of that label; and what frontliners appear to have experienced is a violation of that contract whenever there have witnessed instances of gatherings, non-compliance, or reneging on promises of adequate support and compensation from the legislature. The underlying harm of this sentiment was observed in many countries, as depicted in the image from Spanish artist Luis Quiles in Figure 1.

**Figure 2:**
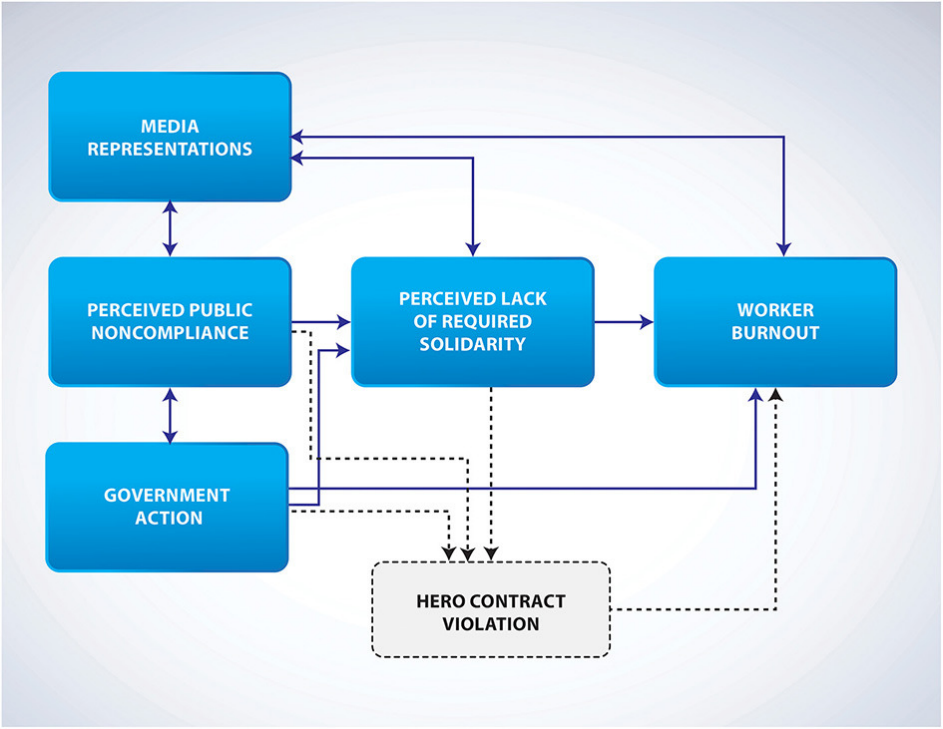
The social contextual factors that contribute to burnout via solidarity appraisal.

The consequences of labelling frontline workers as heroes may also have inadvertently led to a shift in group behaviour where the responsibility for taking action to suppress the virus moved away from the larger collective (the public) to smaller subgroups (frontline heroes). A shift in this sense of responsibility (in light of those more, or uniquely, qualified to take the lead) may also have reduced compliance with public health measures due to the heightened sense of psychological safety and protection that heroes provide to others, reducing the sense of threat (Kinsella et al., 2015a). The failure of the collective efforts to minimise or drive down transmission potentially prolongs the pandemic, and the absence of adequate social support through prolonging the separation of frontline workers from their loved ones, feeding into the burnout concepts of *workload* and *control* (Maslach and Leiter, 2008). This shift in responsibility away from the public may have a direct, negative impact on frontline workers (by increasing morbidity and mortality rates) and also, an indirect, negative impact by reducing the sense of collective and co-operative action to suppress this major health threat. Taken alongside the aspects of media representations and government rhetoric reducing perceptions of solidarity, the combined facets contribute to a unique perspective on occupational burnout in helping professions (Figure 2).

## Toward a Theory of Solidarity Appraisal

In the literature, solidarity has been posited to provide a means of interpersonal coping during times of stress, on the very local (family) level (Knight and Sayegh, 2009), as well as a more widespread (community and cultural) level (Ku and Wang, 2004) during times of crisis. Collective coping as a concept contains within it the ideas of solidarity, recognising that individuals find coping resources both within themselves and through others in their social circles and communities (Pennebaker and Harber, 1993). Yet, perceived solidarity has not yet been considered as an influencing factor on burnout.

It is possible that helping professionals, that we have classically assumed to be associated with altruism (Wakefield, 1993), are perhaps instead inspired partly by a sense of social solidarity (being defined as an empathic response to a condition affecting others: Arnsperger and Varoufakis, 2003). Equally, motivations within helping professionals may be oriented around wider and more complex values or feelings of duty. The concept of *moral capital*, defined as internalised social norms conferring to moral obligation (Silverstein et al., 2012), and that of *social responsibility*, defined as the obligations of those to whom others rely for their well-being (Berkowitz and Daniels, 1964), may also be intertwined in the motivations of the frontline worker.

The present perspective provides an overview of an emerging element of occupational stress present from the findings from the CV19 Heroes project. Participants working in frontline roles of all sectors have spoken of the unique challenges brought about by the pandemic, and their reliance on collective action to keep going. To our knowledge, this theory of solidarity appraisal is the first work that has incorporated socio-cultural factors that exist beyond the immediate workplace in understanding trajectories to burnout. The relevance and applications of this theory will exist beyond the pandemic setting in other roles and contexts where dynamic interplay between the working life of the individual and their social community context exist.

This new position will be of particular relevance to roles or situations where outcomes are linked to social behaviours and collective action, and may also help to understand why communities in different countries around the world have responded to the virus in different ways at varying points in time. For instance, both in the UK and Ireland there were many examples during the first lockdown of people acting in solidarity in both word and deed. The sense of working toward the same goal of virus suppression was evidenced in comparatively limited examples of breaches to public health advice in those early weeks. However, this changed over time, with many notable cases of others abandoning public health advice, and/or being incongruous with appreciation and action to suppress the virus (Fancourt et al., 2020; Faulkner, 2020). Throughout the pandemic, there is an interdependence of action required for those who help to help effectively, and for those who are being helped to therefore benefit most. Solidarity by definition requires reciprocity or it cannot function (Bolle and Kritikos, 2006).

The theory of social solidarity (or lack thereof) underlines the need for responsibility within the media, and from those that moderate and devise community standards for social media. For the legislature there is clear need to communicate with the language of solidarity rather than divisiveness, and to reinforce and celebrate the collective actions toward the common goal in a timely and unified manner (Templeton et al., 2020). It has been noted that the UK government did lead with themes of collective action in their public health advice, however the delay and indecision around the implementation of key measures served to undermine public trust, leaving the message (and perhaps its underlying sentiment of solidarity) weakened (Doogan et al., 2020). Ending the pandemic should reasonably be a shared goal throughout societies, and here the ability to work toward that shared goal could be empowering (with regard to solidarity, broad social support, and maximising personal control), particularly where there is equity of effort and a shared concept of how to reach that goal. This itself is not a new concept, and has been recognised as having huge potential in collective effort as exemplified in the *Blitz Spirit* of the Second World War (Furedi, 2007).

For organisational, social, and cultural psychology there is a need to understand more about what creates a sense of solidarity across a population, and what can protect against the harmful effects of its absence during such challenging times. It will never be possible to have an entirely unified social response (behaviourally or emotionally) during a crisis, and so understanding what can be done to buffer against the harmful effects of needing to press on despite the cumulative hardships that prolonged helping behaviour can incur without broader solidarity is of importance for future crises, but also in other less extreme circumstances. It is currently not clear whether the impact of this lack of solidarity on burnout is through psychological-emotional impacts, or whether through direct impact of increasing their workload, or both; and this will be of importance particularly when considering the extension of this work beyond the pandemic context.

## Conclusion

Perceived solidarity is consistent with, and complements, existing theoretical work on occupational stress, but also offers a useful framework for integrating this work with general social and cultural psychological theory. The social behaviour of the community has impacted frontline workers directly (by increasing infection rate, and therefore workload) but also, indirectly through this social channel where individuals felt betrayed and frustrated as a result of perceptions of the community failing to live up to the social obligations to which they tacitly signed up for by hailing these workers as heroes, or by otherwise hindering the collective effort. These concepts are particularly interesting from a psychological perspective and have application beyond the pandemic setting, particularly in cases where occupations are directly impacted by interdependence of actions and sustained engagement by multiple stakeholders—this is the focus of our forthcoming work.

## Data Availability Statement

The data analysed in this study is subject to the following licences/restrictions: Anonymised data may be made available at OSF on the conclusion of the project subject to participant approval. Requests to access these datasets should be directed to Rachel C. Sumner, rsumner@glos.ac.uk.

## Ethics Statement

The studies involving human participants were reviewed and approved by University of Limerick Research Ethics committee, approval number: 2020_03_52_EHS ER. The patients/participants provided their written informed consent to participate in this study.

## Author Contributions

RS and EK were responsible for the development and writing of this perspective article.

## Conflict of Interest

The authors declare that the research was conducted in the absence of any commercial or financial relationships that could be construed as a potential conflict of interest.

## Footnotes

^1^The CV19 Heroes project was established to track the welfare of workers on the frontline of the pandemic in the UK and Ireland, encompassing all forms of frontline workers from health and social care, to community supply chain, and in civil defence. Project registration can be found at: https://osf.io/nm83c/registrations.

^2^For instance, a doctor can offer professional advice, but the patient must ensure that medication and treatment regimens are adhered to.
